# Coronavirus disease 2019 and stroke

**DOI:** 10.1177/15910199211035920

**Published:** 2021-10-20

**Authors:** Thabele M Leslie-Mazwi, Kittipong Srivatanakul

**Affiliations:** 1 Department of Neurology, 7284 University of Washington, USA; 2 Department of Neurosurgery, Tokai University School of Medicine, Japan

**Keywords:** Severe acute respiratory syndrome coronavirus 2, coronavirus disease 2019, stroke

## Abstract

The multisystem nature of coronavirus disease 2019 has become increasingly clear over the course of the pandemic. Both the neurological and vascular systems are affected, impacting acute stroke. This impact can be conceptualised as direct and indirect effects of the disease. The direct effects of coronavirus disease 2019 on stroke are thought to relate to receptor-mediated tissue invasion and the marked inflammatory response to the presence of the virus. These effects include coagulopathies, endotheliitis, systemic inflammation and atherosclerotic plaque instability, with possibly long-term cardiovascular effects. The indirect effects impact all aspects of stroke care delivery. These extend far beyond the direct effects of coronavirus disease 2019, and represent an essential focus for stroke systems of care. In this article, we detail the impact of coronavirus disease 2019 on acute stroke.

## Introduction

The severe acute respiratory syndrome coronavirus 2 (SARS-CoV-2) produces the illness coronavirus disease 2019 (COVID-19).^1^ After reaching pandemic proportions in March 2020,^2^ it has become increasingly clear that the disease will continue to affect the human population in many ways in the months and years to come. Though predominantly respiratory, increasingly the infection is appreciated as a multisystem disease. Both neurologic and vascular involvement has been reported, and appropriately concern has been high that the disease has an impact on acute stroke. This impact is both direct and indirect.

### The direct impact of COVID-19 on stroke

Multiple mechanisms are likely to be at play in the direct impact of COVID-19 on stroke, but are only partially understood. It is now apparent that the angiotensin-converting enzyme 2 receptor is the functional receptor allowing coronavirus to infect humans.^3^ This membrane-bound aminopeptidase plays both a vascular and immunological role, and is highly expressed in cardiac, vascular and pulmonary tissue. This receptor-mediated initial tissue invasion, and then the marked inflammatory response to the presence of the virus, are thought to produce several of the direct effects of COVID-19 on stroke.^4^

As a result of these direct effects, stroke is seen in patients with limited vascular risk factors otherwise, and in age groups that are unusual for ischemic stroke.^5,6^ While there is strong pathophysiological plausibility to this, caution is required in interpreting this risk. Reports of similar findings of stroke in unusual populations are not widespread in other epicentres in China, Europe, and much of the most affected regions in the United States,^7^ surprising given the incidence reported in published positive samples. Publication bias and sample limitations likely play a prominent role.^8^ Despite this caution, though, it seems likely that stroke presentations are directly impacted by the virus. A clinical phenotype characterised by older age, a higher burden of comorbidities and severe COVID-19 respiratory symptoms, appears to be associated with the highest in-hospital mortality after stroke (almost 60%), and large-vessel occlusions (LVOs) are seen twice as frequently as in the non-COVID-19 population.^9^ In a 1527 patient sample from China a combined datapoint of cardiac or cerebrovascular disease was found in 16.4% of the total cohort.^10^ Available data suggests a risk of acute cerebrovascular events alone of between 1.3% and ∼5% in patients with confirmed COVID-19 infection.^11,12^ This includes both documented haemorrhagic and ischemic strokes. Further, these imaging findings are associated with markers of disease severity (the requirement for intensive care admission, intubation and acute kidney injury) that suggest that patients with more severe forms of COVID-19 may be more prone to the direct impact of the disease on their stroke risk,^13^ perhaps by as much as a factor of three.^10^ Several interacting mechanisms are at play.^14^

## Coagulopathy and thrombophilia

A severe coagulopathy is recognised in patients with severe COVID-19. The characteristics of this coagulopathy (are distinct from those seen with sepsis or disseminated intravascular coagulation are marked by elevated D-dimer and fibrinogen levels but minimal initial abnormalities in prothrombin time or platelet count.^15^ Antiphospholipid antibodies have been described, and may further contribute to thrombotic tendencies.^16^ This coagulopathy and prothrombotic tendency raise concern for both venous and arterial side thromboses, and subsequent strokes. Arterial ischemia in these patients can be macrovascular, in the form of LVOs, or microvascular. The possibility of increased LVO stroke has been reported in case series. In a New York City 329 patient cohort, LVO was present in 31.7% of patients with COVID-19 compared with 15.3% of patients without COVID-19 (*P* = 0.001), while small vessel occlusions were comparable.^17^ A total of 27% of LVO presentations to a Paris hospital during the pandemic peak were COVID-19 positive, with limited respiratory symptoms in the patients affected. Impressively, half of patients had LVO in multiple territories simultaneously.^18^ Clot in these patients appears abnormally friable with a reported increased propensity for clot fragmentation during thrombectomy procedures.^19^ tPA is safe for these patients, with possibly even a lower risk of haemorrhagic conversion (consistent with a procoagulant state).^20^ In addition, venous prothrombotic tendencies have been identified as catastrophic cerebral venous thrombosis in young, previously healthy patients with limited precipitating factors.^21^ Haemorrhagic cerebrovascular disease is less common, ∼0.2% of COVID-19 in a recent cohort,^22^ and less well defined. Deranged coagulation parameters, and markedly elevated D-dimer levels are common findings, and microcirculatory dysfunction appears to contribute further, with a common pattern of multifocal subcortical/cortical petechial-type hemorrhages.^23^ These manifestations are more frequently seen in patients that are severely affected in other organ systems by COVID-19.^24^ While reports of aneurysmal subarachnoid haemorrhage also exist it remains impossible with the limited case numbers to determine if concomitant COVID-19 infection led to instability of the aneurysm wall and subsequent rupture.^25^ This challenge holds for all forms of COVID-19 related haemorrhage and stroke in general: making the distinction between characterisation and causation.^22,26^

## Endotheliitis

Pathological specimens in COVID-19 patients reveal the presence of viral elements within endothelial cells, with inflammatory cell infiltration. Associated with this is evidence of endothelial and inflammatory cell death. This induction of endotheliitis, identified in several organs, is presumably as a direct consequence of viral involvement.^27^ In the cerebral vasculature the subsequent impaired endothelial function may lead to detrimental shifts in the vascular equilibrium towards vasoconstriction or a pro-coagulant state resulting in thrombosis (manifesting clinically as brain ischemia and infarction).^28^ Inflammatory cytokines and vasoactive molecules enhance endothelial cell contractility and the loosening of inter-endothelial junctions, predisposing to leakage through the blood-brain barrier, with haemorrhagic changes.^29^ This proposed central role of endothelial cells raises the question of whether vascular normalisation strategies may modify disease features, possibly including stroke risk.

## Inflammation and atherosclerotic plaque rupture

Systemic inflammation secondary to viral infection has been described to increase vascular events and mortality, predominantly for cardiac disease.^30^ Mechanisms in the context of chronic atherosclerotic vascular disease are largely felt to relate to impact on plaque morphology from inflammation, biomechanical stress and vasoconstriction.^31^ Inflammation alone can lead to thinning of the fibrous cap, lipid influx and expansion of the lipid core, all factors that destabilise the plaque.^32^ While details are limited in the COVID-19 population for cerebral ischemic disease there are isolated reports of higher than expected carotid plaque instability, associated with acute stroke.^33^ Importantly, this may happen in patients less severely affected than many COVID-19 patients that are critically ill when they manifest acute stroke symptomatology.

## Compromise of collateral circulation

Collateral circulation maintains tissue viability in the event of acute ischemic stroke, and therefore limits final infarct size. This vulnerable penumbral tissue is subject to fluctuations in blood oxygen and blood pressure. Both these issues are frequent in COVID-19. Hypoxemia can be profound, requiring proning in severe cases, and in COVID-19 is independently associated with in-hospital mortality.^34^ Coronavirus infection further results in monocyte, macrophage, and dendritic cell activation, and widespread IL-6 release that instigates an amplification cascade with increased signalling in many cell types, such as endothelial cells as discussed above. The resulting increased systemic cytokine production contributes to the pathophysiology of severe COVID-19, including hypotension, cardiac arrhythmias and reduced cardiovascular reserve. This systemic milieu is detrimental to the sustenance of tenuous penumbral tissue and may serve to further amplify the impact of acute stroke in this population.

## Co-morbidities and long-term cerebrovascular disease risk

Metabolic syndrome is a cluster of metabolic disorders that can lead to serious health conditions. Established features include three or more of the following: visceral adiposity, insulin resistance, glucose intolerance, endothelial dysfunction, hypertension and dyslipidaemia. Coronaviruses (and other viruses) metabolically engineer host cells by manipulating gene expression and lipid metabolism to allow enhanced viral replication and progeny release while simultaneously evading host immune responses. Viral infection therefore further potentiates metabolic disease severity.^35^ The phenotype of metabolic syndrome is shared by many patients at risk for stroke, which may increase the likelihood of severe disease in this population. Optimal control of these vascular risk factors is imperative for patients infected with or at risk for COVID-19.^36^ Further, the longer-term implications of infection in patients at risk for metabolic syndrome are unknown. After the severe acute respiratory syndrome outbreak survivors demonstrated altered lipid metabolism over a decade later.^37^ Whether similar long-term concerns will exist for the recovered SARS-CoV-2 population is unknown, but if so may have direct implications for future acute stroke risk and vascular health in general.

### The indirect impact of COVID-19 on stroke

While much remains unknown about the direct impact of COVID-19 on stroke, and the proportion of patients affected, the indirect impact is more clear.^38^ There are several challenges for stroke patients during the pandemic. Mandated social distancing and self-isolation may delay discovery of affected patients. For patients with identified symptoms concerns about COVID-19 exposure in the healthcare environment may limit willingness to present for care. During pandemic peaks or surges there may be a significant strain on ambulance transport, and the required precautions taken by all healthcare providers at all points in the stroke chain of care will lead to requisite delays.^39^ These multiple effects may decrease access to treatment for affected patients. Given the immense therapeutic benefit of acute stroke therapies, and the burden of stroke in our communities at large, it remains an imperative for health care systems to ensure that access is maintained for these essential services, despite the range of indirect effects of COVID-19 on stroke.^40^ A variety of indirect effects are recognised.

## Public awareness and activation of EMS

Public awareness of stroke has long been a challenge in the management of acute stroke. The majority of patients potentially eligible for acute therapies are ineligible because of late presentation to care. In addition, historically patients with more mild deficits are slow to seek medical attention.^41^ In the context of the COVID-19 pandemic these observations have remained true. In Hong Kong, for example, the median symptom onset-to-door time was up to 60 min longer, fewer patients with transient ischemic attacks sought hospital treatment, and the proportion of patients arriving within the therapeutic time window of IV r-tPA was significantly lower.^42^ Patients with mild deficits, already slow to seek attention, are even less likely to do so when faced with the concern of COVID-19 exposure in the healthcare environment. Studies have reported a decrease in more mild stroke presentations.^43^ This decrease occurred in regions with low COVID-19 incidence, suggesting that patient concerns about contracting COVID-19 in hospitals was a primary driver, rather than actual hospital congestion.^44^

## Emergency services

Emergency services during the pandemic experienced an overall emergency medical services (EMS) activation decrease, but on-scene deaths and on scene time increases.^45^ The impact of this on available emergent transport to collect patients from their homes or move them between facilities remains to be clarified. Compounding this is the high routine potential exposure risk to EMS providers. This requires a variety of adaptations in the field when assessing an acute stroke patient,^46^ including the use of personal protective equipment, and sending a ‘scout’ into the dwelling to assess the potential for COVID-19 in the home. These requirements add time delay and may limit the effectiveness of the initial evaluation of the patient, not just in assessing disease severity but in obtaining details such as last seen well and family contact information. Finally, patient triage to a centre capable of delivering thrombectomy may undergo reorganisation in systems that take a global approach to their care delivery, requiring frequent EMS adaptation and re-education.

## Thrombectomy capable hospitals

In regions experiencing pandemic surges, there is the distinct possibility of centres that provide thrombectomy level services becoming overwhelmed for capacity due to the intensive care unit (ICU) needs of the severely affected COVID-19 population at large. This limits the availability of these institutions for the care of acute stroke patients that require thrombectomy. In models borrowed from cardiology,^47^ a care system can establish designated thrombectomy sites for patients presenting with evidence of a secure neurologic deficit in the field.^48^ These locations would ensure access to ICU beds, angiography suites, the relevant personnel and equipment for patients with neurologic deficits who may require thrombectomy. Alternatively, hospital administration must commit to ensuring access for this vulnerable population.^40^ Further consideration is the sequential effect of the required precautions that COVID-19 adds to the evaluation of stroke patients. These patients present almost invariably with unknown COVID-19 status and often fragmentary history. Given that acute stroke may be a presenting symptom of COVID-19 precautions are required for all personnel. This process, termed Protected Code Stroke,^49^ adds a significant delay. This delay is further exacerbated for interfacility transfers. Data from France showed a significant increase in delays between imaging and groin puncture, overall (mean 144.9 min in 2020 vs 126.2 in 2019; *P* < 0.001) and in transferred patients (mean 182.6 min vs 153.25; *P* < 0.001).^19^ A recent meta-analysis showed that the number of stroke alerts during the pandemic was 64% of that during the pre-pandemic period, while the number of reperfusion therapies during the pandemic dropped to 69% of that during the pre-pandemic period.^50^ These delays and decreases translate into patient disability. Further delays due to modifications to inpatient processing must be the focus of specific mitigation strategies, as outlined in several societal and regional publications.^40,51–54^

## Acute stroke team health

COVID-19 can have a significant impact on the acute stroke care team. This can be direct in the form of infections within care personal. In centres with limited neuroendovascular specialists, radiology technologists or interventional nurses the loss of a few key members of the team may severely hamper the ability to deliver care. Fortunately available data for the United States suggests that infection rates among providers were low.^55^ This is a testament to effective precautionary measures and an indication that they should continue until better control of the disease globally is achieved. More insidious is the emotional and psychological toll of the disease as teams worry about their personal health, the health of their families and the future of their practice.^56^ This toll may affect the ability of teams to effectively care for the acute stroke patient.

## Research into acute stroke

Finally, COVID-19 has had a direct impact on the ability of the field to study stroke. Recent data have indicated widespread trial disruption. Following COVID-19, enrolment was suspended at 78% of surveyed sites at which advanced trial infrastructure existed. Beyond this, trial quality was compromised with patients missing clinical and imaging follow-up, and many sites reporting pandemic-related protocol deviations. Human trial expertise has also been affected by the COVID-19 pandemic, with reassignment, furloughing or termination of study coordinators.^57^ What the long-term impact is of this on the science and pace of progress in acute stroke remains to be seen.

## Conclusions

COVID-19 has produced healthcare consequences unprecedented in our lifetimes. Stroke, as with other diseases, is affected in both direct and indirect ways. While understanding the direct impact is vital for disease modification and effective treatment of the individual patient, understanding the indirect effects will allow an effective systemic response.^8^ Strong systems of stroke care will enable us to withstand this pandemic and those likely to follow.^58^
